# In vivo two-photon imaging of the embryonic cortex reveals spontaneous ketamine-sensitive calcium activity

**DOI:** 10.1038/s41598-018-34410-x

**Published:** 2018-10-30

**Authors:** Mikhail Yuryev, Liliia Andriichuk, Marcus Leiwe, Ville Jokinen, Aurelie Carabalona, Claudio Rivera

**Affiliations:** 1 Neuroscience Center, Helsinki Institute of Life Science, University of Helsinki, FI-00014 Helsinki, Finland; 2 RIKEN Centre for Developmental Biology, RIKEN, 2-2-3 Minatojima-minamimachi, Chuo-ku, Kobe, 650-0047 Japan; 3 Present Address: Department of Developmental Neurophysiology, Graduate School of Medical Sciences, Kyushu University, Fukuoka, 812-8582 Japan; 4 Aalto University, School of Chemical Technology, 00076 Espoo, Finland; 5 INSERM, Institut de Neurobiologie de la Méditerranée (INMED), Parc Scientifique de Luminy, F-13009 Marseille, France; 6 Aix- Marseille University, UMR S901, Parc Scientifique de Luminy, F-13009 Marseille, France

## Abstract

Prior to sensory experience spontaneous activity appears to play a fundamental role in the correct formation of prominent functional features of different cortical regions. The use of anaesthesia during pregnancy such as ketamine is largely considered to negatively affect neuronal development by interfering with synaptic transmission. Interestingly, the characteristics of spontaneous activity as well as the acute functional effects of maternal anaesthesia remain largely untested in the embryonic cortex *in vivo*. In the present work, we performed *in vivo* imaging of spontaneous calcium activity and cell motility in the marginal zone of the cortex of E14-15 embryos connected to the mother. We made use of a preparation where the blood circulation from the mother through the umbilical cord is preserved and fluctuations in intracellular calcium in the embryonic frontal cortex are acquired using two-photon imaging. We found that spontaneous transients were either sporadic or correlated in clusters of neuronal ensembles at this age. These events were not sensitive to maternal isoflurane anaesthesia but were strongly inhibited by acute *in situ* or maternal application of low concentration of the anaesthetic ketamine (a non-competitive antagonist of NMDA receptors). Moreover, simultaneous imaging of cell motility revealed a correlated strong sensitivity to ketamine. These results show that anaesthetic compounds can differ significantly in their impact on spontaneous early cortical activity as well as motility of cells in the marginal zone. The effects found in this study may be relevant in the etiology of heightened vulnerability to cerebral dysfunction associated with the use of ketamine during pregnancy.

## Introduction

Although the teratogenicity of exposure to anaesthetics during embryonic development in humans is under debate a consensus is that an increased vulnerability to cerebral dysfunction is associated with exposure to multiple anaesthetics^1^. In rodents and primates though exposure to anaesthetics during early development and perinatal stages has clear detrimental effects even at sub-anaesthetic doses as well as exposure for a brief period of time. E.g. maternal exposure to ketamine during mid-gestational stages leads to abnormal behaviour including cognitive impairment^2,3^. This effect could be derived from changes in network reorganization. Indeed, it is largely accepted that spontaneous early-form cortical activity preceding sensory experience plays an important role in the correct formation of immature cortical neuronal networks. Proper cortical development requires coordinated intracellular and extracellular signaling^4,5^. Calcium fluctuations are critically involved in these processes in the form of independent intrinsic, chemically regulated oscillations as well as coordinated cell population activity^6^. The importance of the intracellular calcium transients in cortical development can be appreciated from studies showing that disruption in calcium homeostasis might affect various brain functions and results in pathological conditions^7–10^.

Early neuronal activity represents distinct intracellular calcium fluctuation patterns^8,9,11–13^. They are known to have a regulatory role in important events of brain development during neuronal proliferation, differentiation and migration^6,14,15^. However, so far calcium activity in the mammalian embryonic cortex has only been investigated under *in vitro* conditions. To our knowledge, only two studies reported *in vivo* calcium imagining in embryos although in conditions where the embryos were isolated from the mothers^16,17^. Importantly, the observations of different patterns of spontaneous activity under *in vitro* conditions do not imply that these exist *in vivo* and as such could be a model of pathophysiological processes. In addition, there is no certainty that the spatio-temporal characteristics of activity *in vitro* will truly reflect *in vivo* conditions. Thus, considering the proposed coding function of intracellular calcium activity on gene expression and neuronal function^18^, the characterization of the properties of spontaneous calcium activity *in vivo* is crucial.

Cellular motility is essential for proper neuronal migration during corticogenesis as well as the incorporation of immature neurons into developing networks. *In vitro* work showed that modulation of calcium influx through NMDA receptors directly affects the coordinated activity in neuronal domains^19^ as well as motility in the developing mouse brain^14,20^. Disruption of these events leads to a detrimental impact on brain development^21^ and can result in a number of developmental brain disorders^22^. The role of NMDA receptors on neuronal migration has been vigorously studied in reduced models, such as dissociated cells from embryonic murine cortex and rat tissue explants^23^. As ketamine is a non-competitive antagonist of NMDA receptors, studying the impact of ketamine on cell motility in the cortex is highly relevant as well as its relation to network calcium activity.

We previously found that Fluo-4AM loading in the *in vivo* embryonic brain is more pronounced in non-proliferating cells of the marginal zone. In this study, we further characterized the cell population and found it to be composed largely of Reelin-positive cells. A subpopulation of loaded cells that were electroporated at E12 with a vector carrying mCherry displayed indeed more mature morphology. Using this approach, we show for the first time spontaneous cortical calcium activity *in vivo*, in a paradigm where the mother provides both the brain oxygenation and nutrition. Thus, this is the closest to physiologically normal conditions ever reported in the mammalian brain. Furthermore, we were able to follow both spontaneous calcium activity and cellular motility - including somatic translocations and fine protrusion motility. Interestingly, these processes were largely unaffected by the use of maternal isoflurane anaesthesia. Strikingly, we found that acute maternal ketamine anaesthesia strongly blocks spontaneous calcium activity and significantly reduced diverse forms of cellular motility *in vivo*. These results show that anaesthetic compounds can differ significantly in their impact on spontaneous early cortical activity as well as motility of cells in the marginal zone. The effects found in this study may be relevant in the etiology of heightened vulnerability to cerebral dysfunction associated with the use of ketamine during pregnancy.

## Results

**Figure 1:**
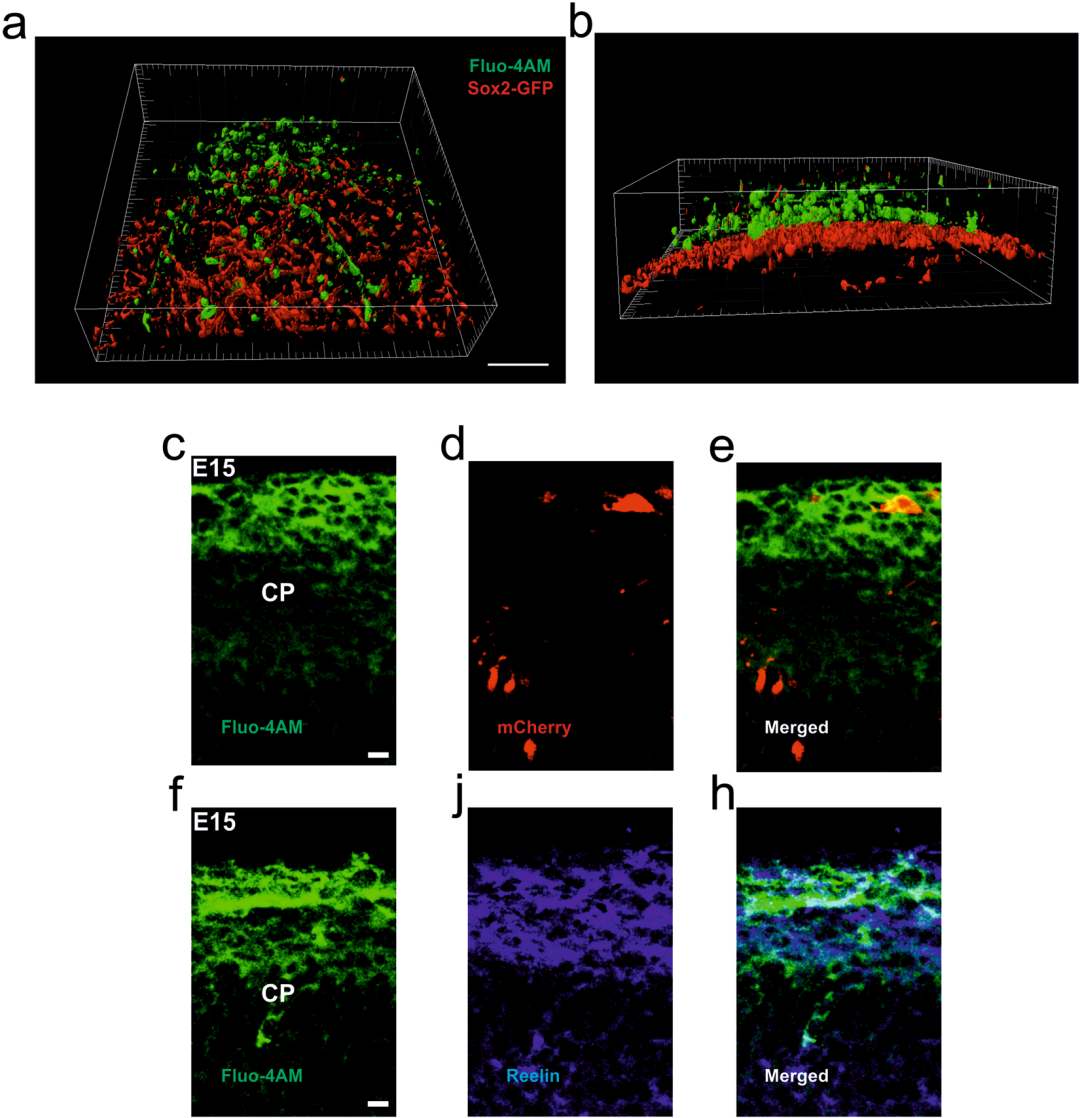
Selectivity of the Fluo-4AM loading to cells in the cortical plate. (**a**,**b**) Orthogonal view of 3D reconstruction of a z-stack from Sox2-GFP (shown in red) reporter embryo loaded with calcium dye Fluo-4AM (shown in green). Z-stack is acquired with an axial step of 3 µm. Scale bar corresponds to 100 µm. (**c**–**h**) Cortical coronal sections from E15 embryos after intraventricular injection of Fluo-4AM (shown in green) (**c**–**e**) Embryos were electroporated at E12 with ubq-mCherry (shown in red) (**f**–**h**) Immunohistochemistry for Reelin marker (shown in blue). Scale bars correspond to 20 µm.

We recently developed an *in vivo* two-photon method to image calcium activity in the mouse embryonic cortex^24,25^. We previously showed that there is a remarkable selectivity of the loading of the Fluo-4AM dye for cell populations closer to the cortical plate. 3D reconstruction from an *in vivo* two-photon-image stack of knock-in transgenic animals that express GFP under the promoter for the neuron progenitor marker SOX2 clearly shows that loaded cells are negative for this marker (Fig. 1a,b). Thus, the apparent preferred cell populations imaged in this study consist of cells at more mature post-mitotic developmental stages^26^. In order to further characterize the Fluo-4AM loaded cell population at E15, we electroporated E12 embryos with a plasmid carrying mCherry. At E15 embryos were subsequently *in vivo* loaded with Fluo-4AM. mCherry expression was found in radially migrating neurons as well as neurons at the marginal zone resembling the morphology of Cajal-Retzius cells. Fluo-4AM loading was found to be strong in the mCherry positive cell population displaying more mature morphology and weak in radially migrating cells (Fig. 1c–e). A characteristic feature of Cajal-Retzius cells is the expression of the extracellular protein Reelin. Indeed, we found a typical pattern of expression at the neocortical marginal zone that strongly colocalised with Fluo-4AM loaded cells in immunostained sections against Reelin (Fig. 1f–h). These results show that there is a remarkable preference of cells of the marginal zone to take up and process Fluo-4AM. This also implies that the calcium activity recorded in this study originates largely from the Reelin-positive cell population of the neocortical marginal zone.

**Figure 2:**
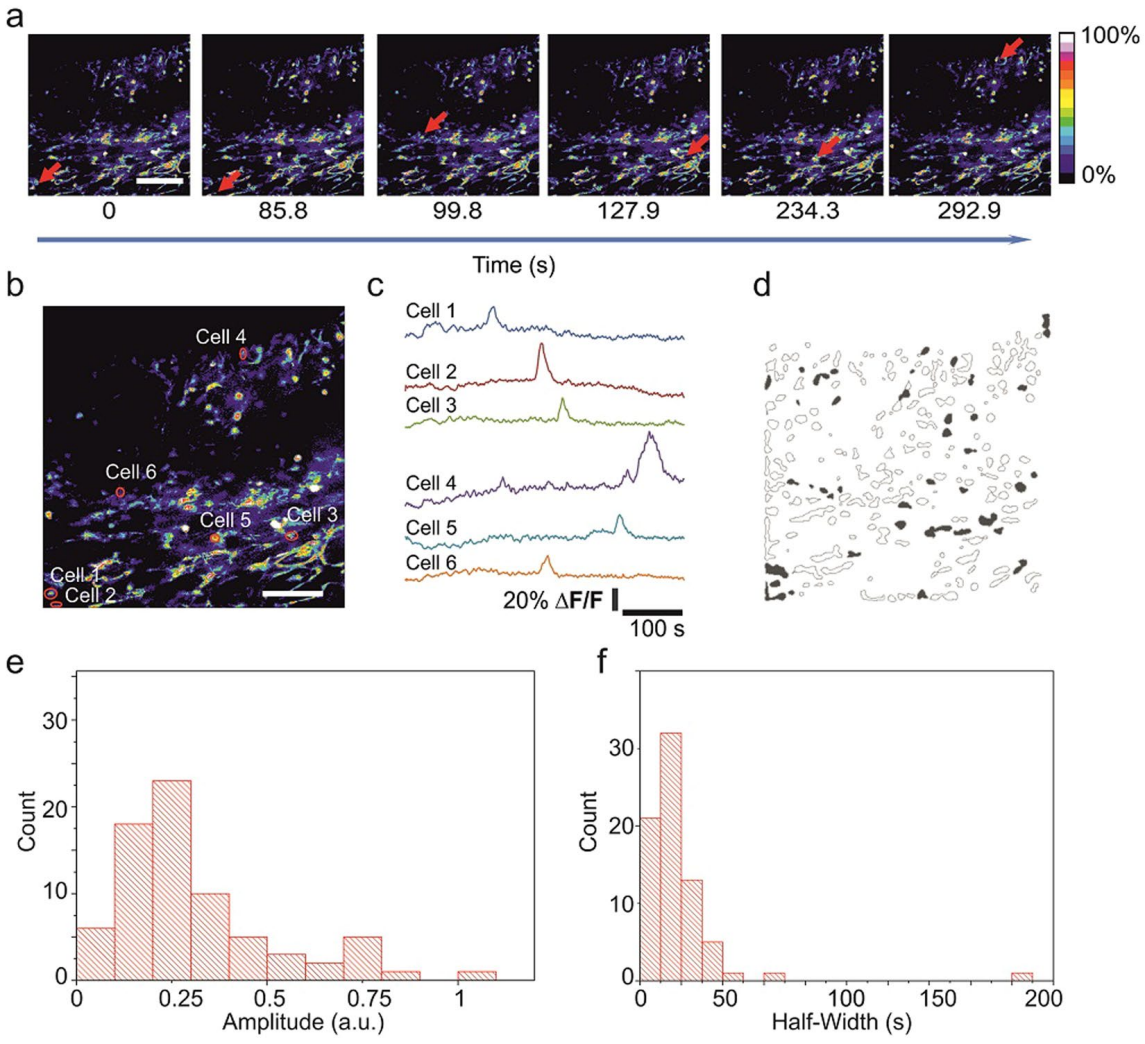
*In vivo* cortical 2-photon calcium imaging of embryos connected to the mother shows spontaneous activity. (**a**) Time-lapse images of the Fluo-4 fluorescence (ΔF/F values). Arrows point to active cells. (**b**) Examples of identified loaded cells are delineated on the left panel and the corresponding fluorescence intensity traces are shown in (**c**,**d**) Filled contours represent active cells during a 500 s recording. Scale bar corresponds to 100 μm. (**e**) Distribution of the amplitudes of calcium fluctuations. (**f**) Distribution of the widths at half-maximum amplitude of calcium fluctuations.

**Figure 3:**
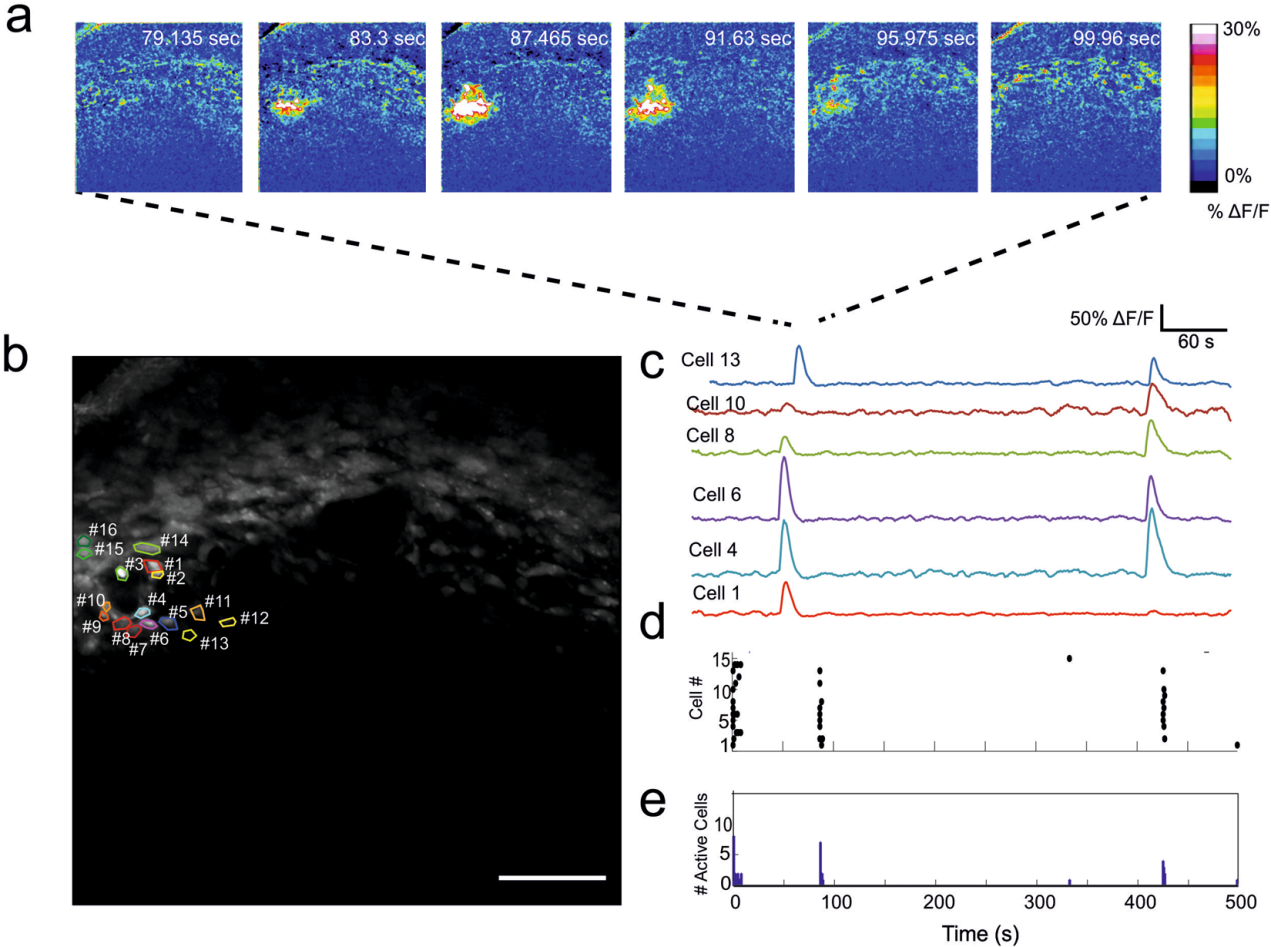
Localised synchronous activity starts in the vicinity of blood vessels. (**a**) Representative time-lapse of ΔF/F images displaying localised synchronous activity. A wave spreading from the initial focal point towards the cortical plate is clearly visible. (**b**) Maximum intensity projection of the time-lapse in a. Coloured ROIs show active cells around a blood vessel. Scale bar corresponds to 100 μm. (**c**) Traces displaying calcium activity of corresponding ROIs in b show no apparent synchronicity. (**d**) Raster plot of Ca^2+^ spikes for all the labelled cells in b. (**e**) Corresponding plot showing the total number of cells active within each frame.

We previously demonstrated that it is possible to follow elicited correlated activity in the form of calcium waves^24^. Presently, we have further developed this method to monitor spontaneous calcium activity in the live embryonic cortex. Apart from improvements in the loading and imaging protocol (see methods) a major change was the use of isoflurane anaesthesia. The changes in the anaesthesia protocol allowed us to monitor and characterize spontaneous intracellular calcium activity in the cortex of embryos at E14-E15 stage of development (Fig. 2a–d, Supplementary Video 1). In a typical experiment around 20% of the imaged cells (66 cells out of 209 detected) were spontaneously active and displayed a bell shape waveform (Fig. 2a–d). The majority (57%) of these calcium events were within the range of 10–30% ΔF/F increases in fluorescence (Fig. 2e). The event-width was measured at the half-amplitude point and ranged from 10 to 30 s for 60% of the observed calcium spikes (Fig. 2f). Within the active pool, we observed various patterns of calcium activity, including propagating calcium waves. However, these patterns of activity were not observed in all the recordings (5 waves detected in recordings from 10 embryos in 7 dams). The number of cells involved in the wave within the imaged area ranged from 10 to 14, with a mean propagation speed of 58 ± 25 μm/s and a mean wave propagation distance 98 ± 14 μm. Analysis of areas that display synchronous activity seemed to suggest that limited local propagation occurs (Fig. 3a). With nearby cells exhibiting very similar patterns of calcium transients (Fig. 3c), while distal cells populations do not show synchronized calcium transients. Analysis of raster plots and summation plots also showed a high degree of synchronicity within the proximal cluster in these events despite their sparse nature (~1 event every 5 minutes; 0.0033 Hz) (Fig. 3d and e respectively). A closer analysis of the propagation indicates the possibility of a spatio-temporal component to these events. Spike Timing Tiling Coefficient (STTC) analysis shows a higher degree of correlation to nearby cells than to those further away, even though they still contribute to the pattern (Supplementary Fig. 1a,b).

**Figure 4:**
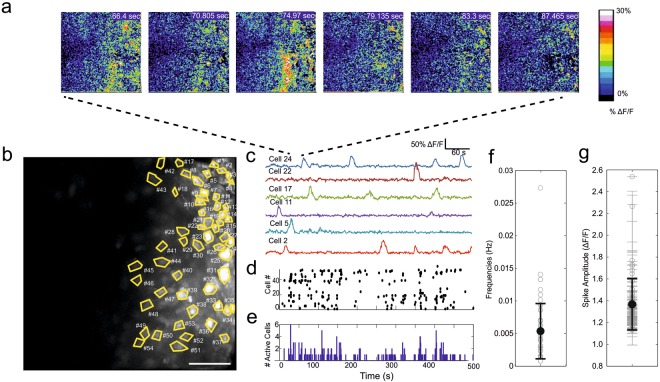
The most common type of activity displayed by embryonic cortex was in the form of non-synchronous spiking. (**a**) Representative time-lapse of ΔF/F images with stochastic activity. (**b**) Maximum intensity projection of the time-lapse in a. Scale bar correspond to 100 μm. (**c**) Representative traces which display a high degree of variability in the timing of calcium transients. (**d**) Raster plot of Ca^2+^ spikes for all the labelled cells in b. (**e**) Corresponding plot showing the total number of cells active within each frame. (**f**) Average frequency of spiking activity. The data are presented as the mean ± SD. (**g**) Average spike amplitude. The data are presented as the mean ± SD in bold, thinner error bars represent SD for each individual cell.

A second more common pattern of activity was apparently randomly distributed (Fig. 4) appearing in all recorded embryos with spontaneous activity (10 embryos in 7 dams) with a broad range of single cell frequency of activity (5.4 ± 4.2 × 10^−3^ Hz; Fig. 4f). The cumulative calcium event amplitude also displayed a very large distribution at the population level (amplitude 13.7 ± 2.3% ΔF/F) and within a single cell (average standard deviation of amplitude within single cells: 12% ΔF/F; n = 66 Fig. 4g). STTC analysis of transients in embryos displaying apparently stochastic activity did not show a significant spatio-temporal component (Supplementary Fig. 1c,d).

NMDA receptor signalling has been shown to be involved in the mechanism for spontaneous activity of cells in the embryonic cortex *ex vivo*^14,27,28^. The drug ketamine is a strong inhibitor of NMDA receptors signalling. Although it is a common anaesthetic compound, neither its acute effect on the inhibition of NMDA signalling nor its effect on spontaneous recurrent activity in the embryonic cortex *in vivo* has been shown previously. In order to address this question, we used the anaesthetic ketamine delivered either by ventricular injection or to the mother.

**Figure 5:**
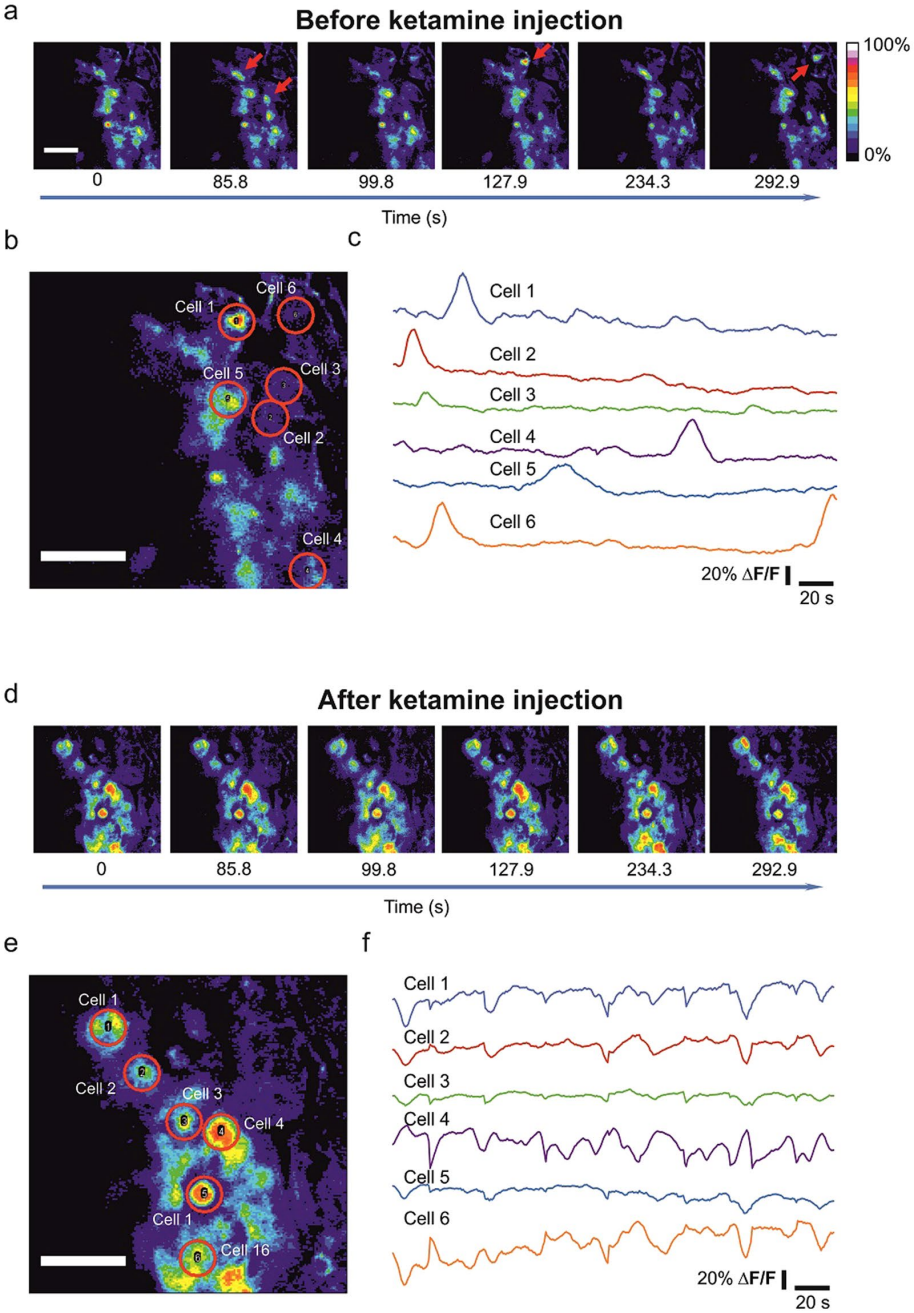
The effect of intraventricular ketamine application on *in vivo* calcium activity. (**a**) Time-lapse images of Fluo-4 fluorescence absolute ΔF/F values before injection. (**b**) Red circles are regions of interest on identified loaded cells in a. Scale bars correspond to 50 µm. (**c**) Fluorescence intensity traces in the identified cells in b. (**d**) Time-lapse images of Fluo-4 fluorescence showing the ΔF/F values after intraventricular injection of 50 mg/ml ketamine. (**e**) Red circles are regions of interest on identified loaded cells in d. Scale bars correspond to 50 µm. (**f**) Fluorescence intensity traces in the identified cells in e. The slight variations in the signal are rising from the motion artefacts, as could be appreciated from synchronicity of the disturbances.

**Figure 6:**
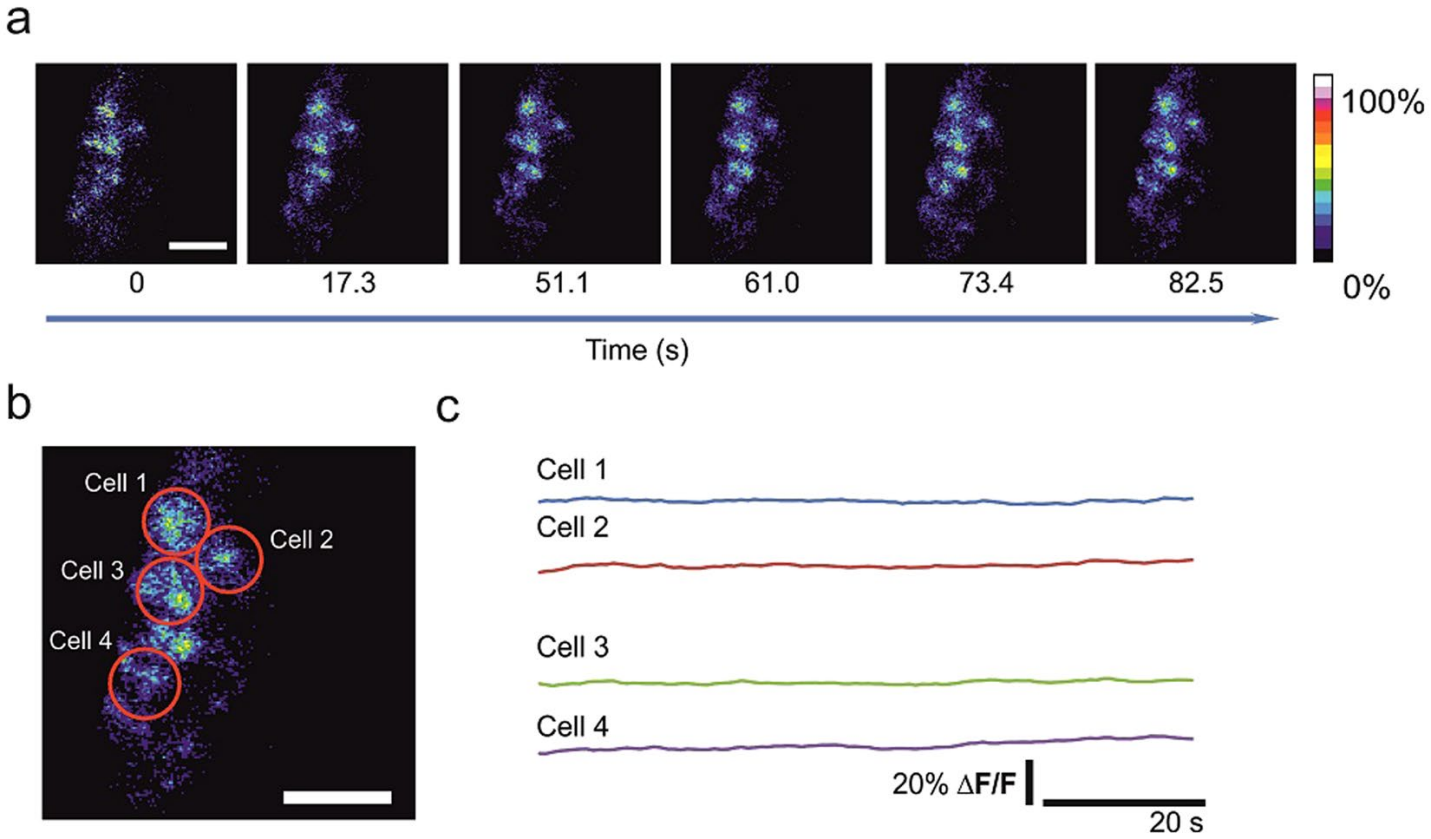
The imaging of calcium activity under maternal ketamine/xylazine anaesthesia. (**a**) Time-lapse images of the Fluo-4 fluorescence ΔF/F values in embryos from dams anesthetized with ketamine/xylazine. (**b**) Red circles are regions of interest on identified loaded cells in a. (**c**) Fluorescence intensity traces in the identified cells in b. Scale bars correspond to 50 μm.

For this purpose, we first slowly injected 1 µl (50 mg/ml) ketamine intraventricularly to the embryonic brains. We estimated this amount to match the concentration reached in the embryo during the application of anaesthesia to the mother during common surgical procedures on mice *in utero*^24^. Indeed, ketamine application caused a strong reduction in calcium activity (n = 5 embryos from 4 dams) (Fig. 5, Supplementary Videos 2 and 3) in contrast to the vehicle application (n = 2), which did not produce any significant effect (Supplementary Video 4). This result suggests that ketamine anaesthesia delivered to the mother could acutely influence the spontaneous calcium activity of the embryo. In order to investigate this, we next imaged calcium activity in the embryonic cortex while the mother was anaesthetised with a ketamine/xylazine mixture (80/10 mg per kg of weight, respectively), which is routinely used in animal experiments. It is important to point out that in the method used here the mother-embryo interface has a fully functional placental barrier. In agreement to the results obtained with our intraventricular injections, we did not observe spontaneous activity under maternal ketamine/xylazine anaesthesia (Fig. 6; the corresponding baseline before ketamine recording is shown in Fig. 2c)^24^. This confirms the diffusion of ketamine through the placental barrier and discloses a strong acute effect on *in vivo* embryonic cortical activity.

**Figure 7:**
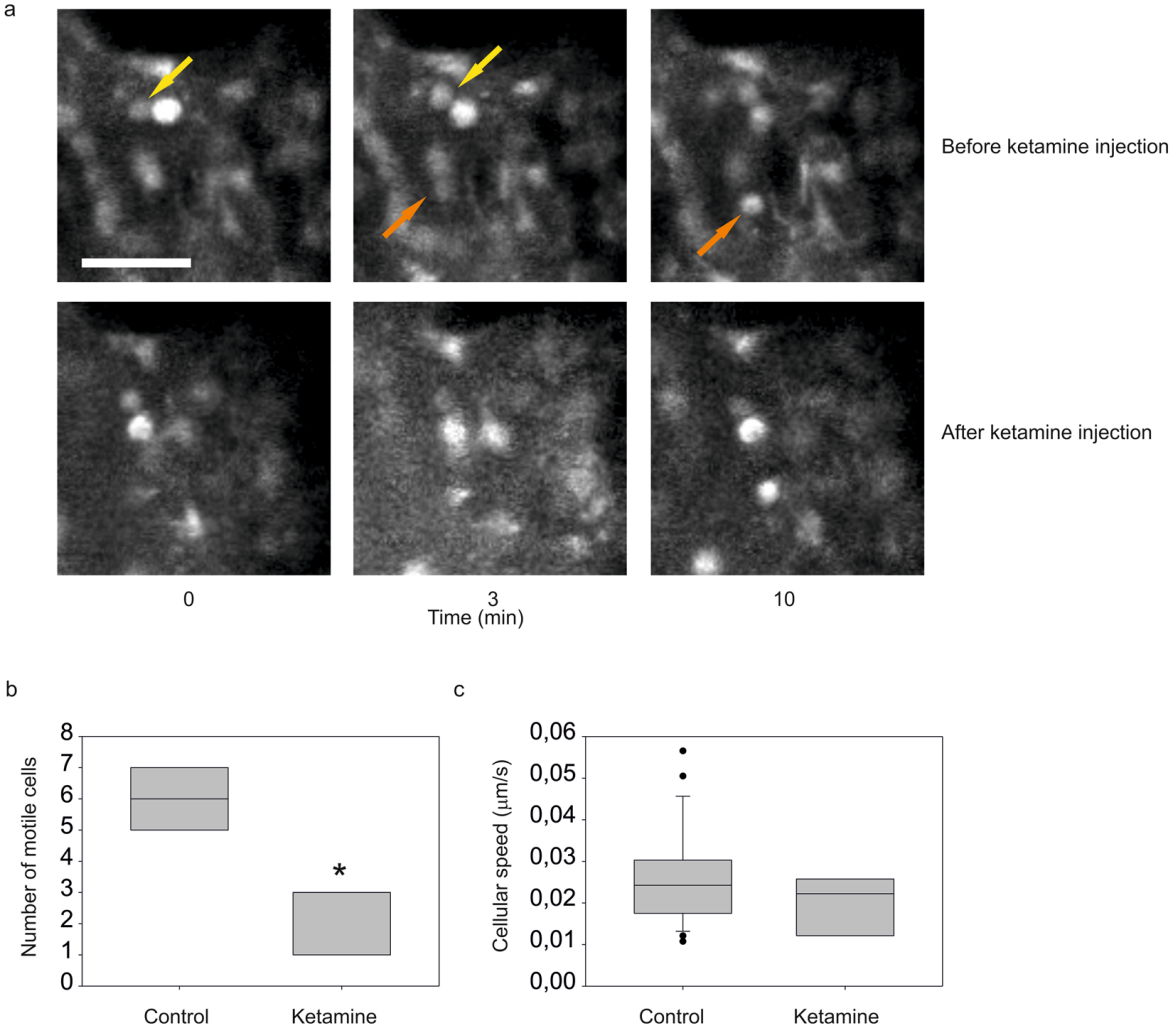
The number of migrating cells is reduced by acute ketamine anaesthesia administration. (**a**) Representative images of the same area with moving cells before (upper plane) and after (lower plane) ketamine injection. Ketamine application blocks the motility of the cells. Scale bar corresponds to 50 μm. (**b**) Quantification of cells showing somatic translocation. The asterisk represents statistical significance (p = 0.03), Mann Whitney nonparametric test. (**c**) Cellular speed measurements by somatic displacement. Boxes show the median and the interquartile range.

Apart from the importance of monitoring network activity, calcium transients are known to be tightly connected to neuronal motility in embryonic brains^14,15,29^. In recent studies, *ex vivo* slices have defined in more detail the role of the calcium-mediated communication on a cellular motion. Especially, the fact that the somatic translocation of radial glia appears to be correlated with bursts of calcium activity. While during periods of rest, calcium activity is significantly lower^17^. Apart from an effect on intracellular calcium activity, NMDA receptor blockade has been demonstrated to decrease the motility of migrating cells^14^. In the next set of experiments, we ask whether ketamine administered to the mother could acutely affect cell motility in maturing cells of the cortical plate. In control conditions (Fig. 7a, upper plane) a subpopulation of Fluo-4AM loaded cells in the cortex are highly motile (Supplementary Video 5). Different populations of cells are engaged in different forms of motility, which most likely represents different stages of maturation.

Following the injection of ketamine (n = 3 from 2 dams) (Fig. 7a, lower plane) cellular motility was decreased in general (Supplementary Video 6). A fraction of cells displayed somatic translocation. This population was significantly reduced in the presence of ketamine (before: 5.7 ± 0.4; after: 2.0 ± 0.58; Mann Whitney test P = 0.03, n = 4 embryos; Fig. 7b), with a slight non-significant reduction in the somatic translocation speed (before: 0.024 ± 0.01 mm/s, n = 23 cells; after: 0.022 ± 0,019 mm/s, n = 8 cells in 4 embryos; Fig. 7c). These results imply that the migration motility of a subpopulation of cells is sensitive to ketamine and the speed of the non-sensitive population is not correlated with the general effect of ketamine on calcium activity.

**Figure 8:**
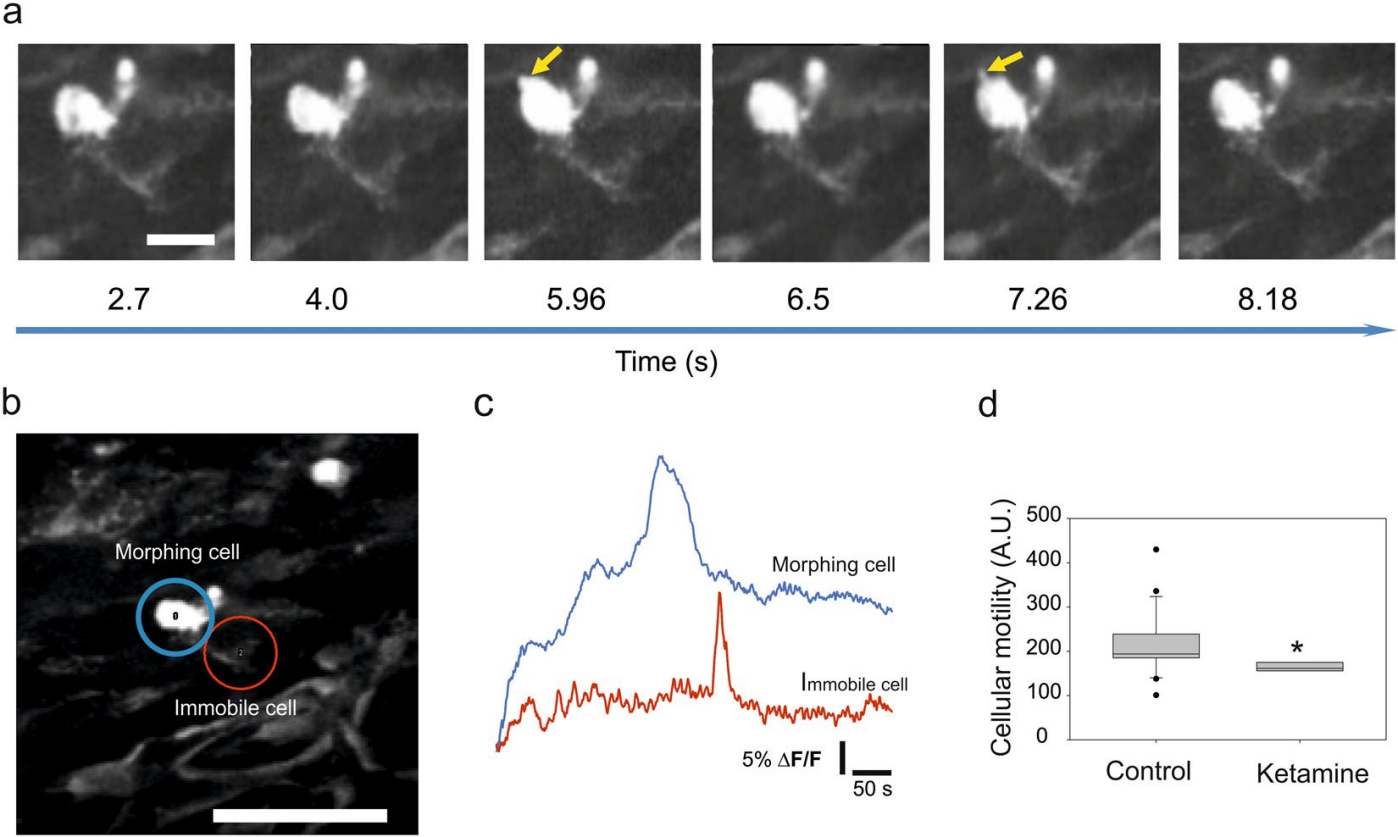
Cells with protrusive motility display faster calcium kinetics and are sensitive to ketamine anaesthesia. (**a**) Time-lapse of a cell showing protrusive motility. The arrow points to one of the dynamic protrusion during the recording. Scale bar corresponds to 25 μm. (**b**) Regions of interest marking cell with protrusive motility (blue circle) and a non-morphing cell (red circle). Scale bar corresponds to 50 μm. (**c**) Fluorescence intensity traces in the regions of interests from b. (**d**) Cellular protrusive motility measured by pixel displacement before and after ketamine anaesthesia. The asterisk represents statistical significance (p = 0.01), Mann-Whitney nonparametric test. Boxes show the median and the interquartile range.

In the most stable recordings and following filtering it was possible to follow not only the somatic translocation, but also subtler cellular changes. Some cells displayed changes in cell shape in the form of protrusive motilities without soma translocation (Fig. 8a, Supplementary Video 7). The protrusion motility was blocked (n = 3 embryos from 2 dams) by intraventricular application of ketamine 50 mg/ml (Supplementary Video 8). To quantify cellular protrusive motility, we applied the method of pixel displacement, which has been previously used for measuring the protrusive motility of filopodia. This quantifies the pixel displacement as the standard deviation of the intensity^30^. Cellular motility measured by pixel displacement was significantly reduced after ketamine application (before: 213 ± 69, n = 24 cells in 4 embryos; after: 164 ± 24, n = 9 cells in 4 embryos, Mann Whitney test P = 0.01; Fig. 8d).

Moreover, we compared the calcium activity patterns of cells, which are motile or actively produce protrusions and the immobile cells. The waveform of calcium spikes in the immobile cells, which are presumably integrating into early neuronal networks, appeared to be faster and sharper than in the motile cells (Fig. 8b,c). Correspondingly, in the regions of the cortex, which showed a higher frequency of activity, we observed less motile cells than in the regions with slow spontaneous calcium activity. We attempted to correlate migratory motility with calcium activity at the cellular level. Out of 26 cells from 6 embryos only one cell showed a tendency to correlation (data not shown). The lack of correlation could be due to the relatively short time and distance recorded compared to *in vitro* conditions where the correlation between speed and calcium activity has been observed^17^.

Previous reports have shown a strong trophic effect for activity on neuronal survival, where the inhibition of network activity leads to cell death^31^. To test whether the calcium activity inhibited by ketamine causes cell death, we anaesthetised the mothers with ketamine/xylazine and isoflurane for 3 hours and harvested the embryos on the following day. We stained the brain slices for the apoptotic marker caspase-3. Examination of E15 cortical sections immunostained for cleaved caspase-3 did not show any significant activation of this apoptotic marker (Supplementary Fig. 2).

## Discussion

The proper supply of oxygen and chemical cues is crucial for the normal activity in the developing brain. Thus, only the native *in vivo* environment can reflect the full complexity of interactions between blood flow and the active cells. Recent significant advances in the field of two-photon imaging^16,17,24,25,32,33^, allow the imaging of developmental processes while maintaining physiological conditions of live embryos. In the present work, we implement this method for calcium imaging of the cortex in embryos connected to the mother through the umbilical cord. The combination of both physiological conditions and optimized anaesthesia allow for the recording of spontaneous calcium activity. This, to our knowledge, is the first time spontaneous cortical calcium activity has been recorded in live mammalian embryos where the mother-embryo interface is intact.

Although, we managed to make stable recordings and maintained the preparation for sessions that lasted around 4 hours, the success rate for long recordings was very low. A major problem was intrinsic tissue motion artifacts that are mostly derived from embryo heartbeat and other physiological processes. These are challenging to compensate for when scanning only one single focal plane. In our hands, the best-stabilized recordings were usually obtained from tissue located near the holder, which, however, also undergoes the higher stress due to the immobilization procedure. In the future, the introduction of fast three-dimensional scanning^34,35^ will enable the possibility of better image corrections in the z- dimension. This will ensure that more stable, reliable and longer calcium recordings can be performed.

The characteristics of the waveforms and patterns of cellular calcium activity are known to change during embryonic neuronal maturation of the cortex^36–38^. However, whether similar patterns occur *in vivo* is unknown. Our recordings show the existence of spontaneous recurrent activity in the form of synchronized and apparently stochastic intracellular calcium fluctuations. The kinetics of the stochastic events are similar to the ones encountered in slice preparations from late embryonic cortex^36–38^ with the exception that we did not observe the plateau transients that are frequently observed in *in vitro* recordings^36^.

Acetoxymethyl-labelled Fluo-4AM calcium dyes demonstrated a peculiar property to label preferentially differentiated cells, and was not taken up by radial glia and other undifferentiated cells^24^. In the present work, we further characterised this phenomenon to better define the population of loaded cells. In agreement with our previous observations 3D reconstruction of the neocortex of Sox2 reporter mice at E15 showed clearly that Fluo-4AM loading is absent from Sox2-positive cells. The selectivity was further demonstrated in the experiment were mCherry positive electroporated cells that were not engaged in radial migration showed Fluo-4AM loading. A major population of the cells displayed morphology and positional corresponding to Caja-Retzius cells. As this cell population has the characteristic to express the extracellular protein Reelin we tested for colocalisation of the loading with Reelin-positive cells. Indeed, we found a major preference of the unesterified or free-acid form to Reelin-positive cells. These results imply that the recordings presented in this study derived primarily from Reelin-positive cells. As to the cause of the difference in loading pattern between *in vitro*^16,29,39,40^ and *in vivo* we do not have a clear explanation. However, this is not uncommon; other dyes have been reported to show different selectivity between the two conditions e.g.^41^.

In the present study, we found a peculiar form of spontaneous synchronized recurrent activity that engaged cells surrounding the blood vessels. This is noteworthy, as blood vessels are known to regulate the growth and division of cells via various trophic factors. Furthermore, they can also serve as migration guiding posts. Based on previous results^24^ we know that cell populations in the upper layers of the embryonic cortex have the capacity to display large correlated activity similar to calcium waves upon focal stimulation. Interestingly, the number of cells participating in each spontaneous wave in the present study was very limited. As the directionality of the wave was from proximal to distal cells from the vessels, it is plausible that the mechanism for this sort of activity includes a triggering step by a cell closer to the blood vessel that later spreads through gap junctions and to the interconnected cell populations. A likely candidate for the triggering cell populations could be pericytes that reside in the proximity of the blood vessel. In the future, more detailed investigations of the cell populations engaged in spontaneous activity and the mechanism for the start and wave propagation are warranted. To this end, a combination of simultaneous calcium and voltage recordings as recently performed in muscle cell preparations could be helpful in disclosing this mechanism^42^. In addition, the present *in vivo* method can help to study the interaction between the blood vessels and spontaneous activity as well as their importance for developing cortical networks. Importantly, this kind of study is impossible to perform in *in vitro* conditions, which lack the proper blood-flow supply. Furthermore, *in vivo* imaging could reveal the interaction between the forming blood-brain barrier and network activity.

Migrating neurons express functional NMDA receptors at early stages of brain development^27,43,44^. Previous works indicated that NMDA receptors regulate the motility of new-born neurons during both tangential and radial modes of neuronal migration^44^. However, the mechanism of involvement of NMDA-receptors mediated activity^45^ in the cellular motility in embryos is unclear *in vivo*. Here, we show that ketamine blocks calcium activity of embryos in *in vivo* conditions in an acute manner. This is in agreement with the fact that functional NMDA receptors are present and suggest their importance for spontaneous activity in the developing brain. Importantly, the use of ketamine on the mother had a similar acute effect as direct microinjection of the drug to the embryo’s brain. These results indicate that ketamine is rapidly transferred to the embryo’s bloodstream and produced a significant block of cortical calcium activity in the embryo.

Although ketamine affects network calcium activity, one anaesthetic session does not result in the apoptosis activation in contrast to chronic exposure to ketamine^46^. In accordance with these previous results, the use of ketamine in the present study did not result in a significant increase in the expression of the apoptotic marker, cleaved caspase-3. This implies that the effects observed were not the result of the indirect apoptotic effect of ketamine.

Correct motility is crucial to allow developing cells to migrate to their correct location in the cortex and is also essential for network formation and functioning. The duration of imaging used in the present study excludes the monitoring of large cellular displacements but it does allow for the tracking of cellular motility over short migratory distances. Analyses on cell motility in combination with calcium imaging, showed the waveform of spontaneous calcium transients were quite different between immobile cells and cells showing morphing motility. Interestingly, *in vivo* ketamine injection blocked not only calcium activity, but also cellular motility, including the protrusive motility of the cells. These results suggest that the link between the pattern/waveform of calcium transients and cellular maturation is preserved *in vivo* and not a consequence of pathophysiological conditions present in *in vitro* conditions.

Correlated calcium activity in embryonic slices has only been shown to appear *in vitro* in differentiated neurons at the later stages of development (E17-E18)^36^ rather than the stages described in the present work (E14-15). The reported propagating calcium waves recorded in this study suggest that this sort of early activity is critically dependent on a blood supply that is properly oxygenated and carrying nutrients and supplements from the mother (which differed radically from in *in vitro* conditions).

Although the teratogenicity of exposure to ketamine during gestational stages is well documented^1–3^ detailed investigation of the underlying mechanisms *in vivo* is scarce. The results presented here clearly show that acute application of ketamine directly into the embryonic brain has a strong inhibitory effect in spontaneous neocortical activity as well as cell motility. Interestingly, the use of isoflurane maternal anaesthesia did not influence spontaneous activity in the embryos unlike reported in the early postnatal brain. This pronounces difference between ketamine and isoflurane could be based on the relative selectivity of ketamine to inhibit NMDA mediated signalling. The obtained results prompt for further investigation of the role of NMDA signalling in the enhanced propensity to cerebral dysfunction associated with ketamine exposure during pregnancy.

## Conclusions

Using our previously developed method for embryo *in vivo* two-photon imaging, we successfully imaged spontaneous calcium activity in mouse embryos connected to their mother via the umbilical cord. We found a peculiar characteristic of the Fluo-4AM dye to label preferentially upper cortical plate cell and marginal zone including a population of Reelin-positive cells with morphology resembling Cajal-Reztius cells. Along with activity, we were able to simultaneously visualise cellular motility, including somatic translocations, and fine cell protrusions formation. The present work clearly shows that previously found *in vitro* patterns of cortical activity are relevant to the embryonic neocortical activity *in vivo*. A particularly interesting finding was the difference in sensitivity to isoflurane and ketamine anaesthesia. The spontaneous activity in the embryonic cortex was not sensitive to isoflurane maternal anaesthesia where we could follow both synchronized and non-synchronized patterns of activity. Whereas ketamine application resulted in strong suppression of calcium activity in general as well as cell motility. These results may be also relevant for the involvement of ketamine in the propensity to abnormal brain function following maternal exposure to this compound during gestation.

## Methods

### Approval

All the experiments involving animal subjects were approved by the National Animal Experiment Board, Finland (license number ESAVI/8672/04.10.07/2015). All methods were performed in accordance with the relevant guidelines and regulations.

### Animals

The pregnant mice at gestational stage E14-E15 were used for the experiments. Female c57bl6/JOla mice paired with Sox2-GFP males of the same background^47^ and female ICR mice paired with GAD67-GFP of the same background^48^.

### Embryo immobilization

Embryo immobilization was performed as described earlier^24^ with the following modifications: the pregnant dam was anaesthetised by isoflurane with 3.5% for induction and 1-1.2% for maintaining anaesthesia. The abdomen was opened and the uterine horn was taken out through the membrane in the cell culture dish and fixed in the custom-made heating plate. The embryo was immobilized and glued to the metal holder.

### *In vivo* two-photon imaging of embryos

Prior to imaging, embryos were injected intraventricularly with Fluo-4AM calcium dye (17 μg of calcium dye Fluo-4AM, Molecular Probes) dissolved in 3 μl of 20% F-127 pluronic**®** acid (Sigma) in dimethyl sulfoxide (DMSO, Sigma) that was then diluted in artificial cerebrospinal fluid (ACSF, in mM: 125 NaCl, 1.25 NaH_2_PO_4_, 2 CaCl_2_, 1 MgCl_2_, 5 KCl, 20 D-glucose, 10 HEPES) to reach a final concentration 0.4 mM of Fluo with 13 mM FastGreen dye (Sigma) added for injection guidance.

Two-photon imaging was performed with Olympus Fluo View 1000MP system at the rate 1.2 fps at a resolution 512 × 512 via 25X objective (XLPlan N, Olympus). Excitation light was produced by Mai-Tai DeepSee laser (Spectra Physics).

### Image processing and analysis

Before filtering, the slices with strong motion artefacts were deleted from the image stacks. Image stacks were further filtered using Kalman filter (plugin for ImageJ written by Christopher Philip Mauer) and descriptor-based registration^49^. Intensity profiles of the cells were analysed with ImageJ and Mini Analysis Program (Synaptosoft Inc.). Image segmentation was performed with the custom-made routine for MATLAB (D. Aronov, T. Thressard, R. Cossart). For speed measurements, the distance between the initial and final position of the cell was divided by the time of the motion. For motility assessments, the standard deviation of the image stack was used to measure the pixel count of the overall occupied space in the image over the recording period. The Student t-test was used for the comparison of normally-distributed groups and the Mann-Whitney rank test was used for the comparison of non-normally distributed groups.

### Network activity analysis. Motion and Drift Correction

Prior to motion correction, frames with extensive motion were removed. Initial coarse registration (translation-only) of image stacks to the first frame were performed using TurboReg^50^. Subsequent motion correction and drift correction were performed by using SPM (www.fil.ion.ucl.ac.uk/spm). Noise removal and subsequent ΔF/F movies were performed using AFID (a MATLAB plugin created by Dr. A. Lowe, http://www.kcl.ac.uk/ioppn/depts/devneuro/Research/groups/lowe.aspx)^51^.

### Spike Detection

Cells were manually selected and ΔF/F values were calculated for each cell. To detect calcium events, a normal distribution was fitted to the lowest 90% of data points. Bursts/Events/Spikes were defined as points that were 3 standard deviations above the mean value calculated from a 10 second window around the time point in question.

### Spike Timing Tiling Coefficient (STTC) analysis

Calculations were performed as described previously^52^. Correlations were counted within a 2 second window (i.e. ΔT = 2 seconds).

### Selectivity of the Fluo-4AM loading to cells in the cortical plate by in utero electroporation and immunostainings

Wild-type ICR mice were paired and a day of vaginal plug was determined as E0.5. Pregnant mice at the gestational day E12 received analgesic buprenorphine 30 min before the surgery (0,03 mg/kg body weight) and anaesthetised by isoflurane with 4% for induction and 2-2,5% for maintaining anaesthesia. The uterine horns were exposed and embryos were injected intraventricularly with 1 µg ubq-mCherry (gift from I.Medina) per embryo combined with Fast Green (2 mg/ml, Sigma). The parameters of electroporation were following: 5 pulses, 50 V, 50-ms interval cycle length, 950-ms intervals. The mice were monitored post-surgically and given anti-inflammatory Rymadil (10 mg/kg body weight). Embryos were exposed and injected intraventricularly with Fluo-4AM calcium dye (dissolved as mentioned above) at E15 and placed back to the abdomen and were collected in 2 hours into ice-cold 4% paraformaldehyde and fixed overnight. 70 µM thick coronal sections were made and immunostained with a primary mouse monoclonal antibody Reelin (Santa Cruz, 1:250) and secondary Cy5 (Jackson ImmunoResearch, 1:200). Images were produced using Zeiss LSM 710 confocal microscope via 20X objective.

### Apoptosis assessment with immunostaining

c57bl6/JOla mice paired with Sox2-GFP males have been used at E14 gestational day. To check the effect of anaesthesia on apoptosis, mice were divided to ketamine, isoflurane and control groups. Duration of experiments was 3 hours. Mice were placed on the heating pad maintained at 37 °C. The depth of anaesthesia was checked by the toe pinch method and also their respiratory pattern. To prevent dehydration, mice were injected via subcutaneously with sterile phosphate-buffered saline 1.5 hours after starting of experiments. The ketamine group was injected subcutaneously with a dosage of ketamine (80 mg/kg body weight) combined with xylazine (10 mg/kg body weight). Mice received 1/2 and 1/4 dosages of ketamine/xylazine mixture in 30 minutes and 2.5 hours respectively after start of the experiments. The isoflurane group was anaesthetised by isoflurane with 4% for induction and 2% for maintaining anaesthesia. Animals recovered for 24 h and then were sacrificed through a CO_2_ euthanasia procedure at E15. Embryos were then fixed in ice-cold 4% paraformaldehyde overnight. 20 µM thick coronal cryosections were made and immunostained with primary polyclonal anti-cleaved caspase-3 antibody (Cell Signalling, 1:300), secondary Alexa 568 (Molecular probes, 1:400) and Hoechst 33342 (Molecular probes, 1:1000). Images were produced using Zeiss Image M1 Colibri system with LED illumination via 20X objective.

## Electronic supplementary material

Supplementary figures

Supplem. Video 1

Supplem. Video 2

Supplem. Video 3

Supplem. Video 4

Supplem. Video 5

Supplem. Video 6

Supplem. Video 7

Supplem. Video 8
