# Supplementary material for: In vivo two-photon imaging of the embryonic cortex reveals spontaneous ketamine-sensitive calcium activity
Source: Sci Rep. 2018 Oct 30;8:16059. doi: 10.1038/s41598-018-34410-x (PMC6207746; doi:10.1038/s41598-018-34410-x)
Supplement: Supplementary file 1 — Supplementary figures [file 41598_2018_34410_MOESM1_ESM.docx]

**In vivo two-photon imaging of the embryonic cortex reveals spontaneous ketamine-sensitive calcium activity**

*Mikhail Yuryev^1^, *Liliia Andriichuk^1^, Marcus Leiwe^2^, Ville Jokinen^3^, Aurelie Carabalona^4,5^ and Claudio Rivera^1,4,5^

Supplementary figures (Suppl. Fig.1-2)

Video legends (Suppl. Video 1-8)


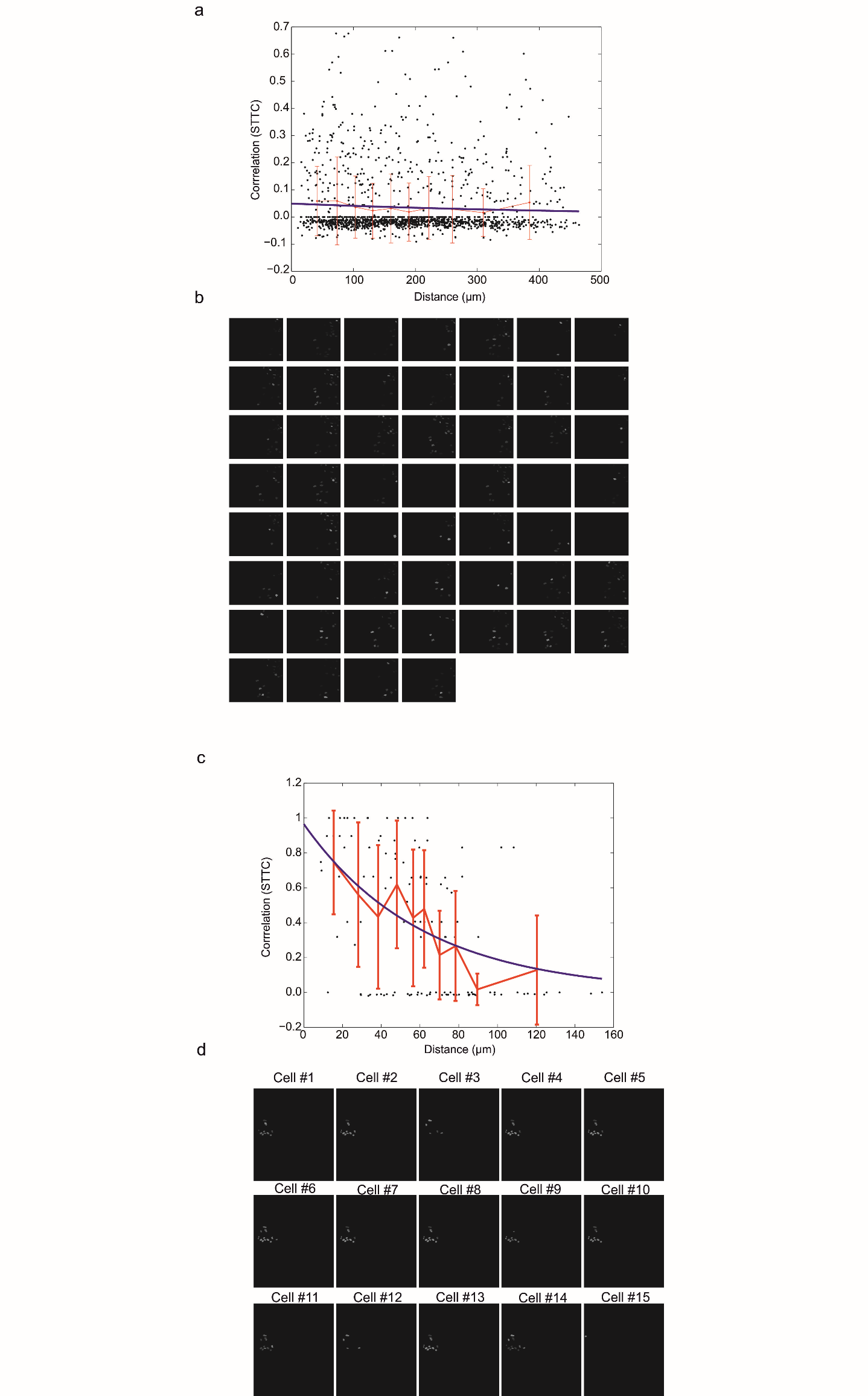


**Supplementary Figure 1.** **Analysis of spatio-temporal events.** Spatio-temporal patterning occurs during synchronous events. (a) The correlation of each cell to all other cells (black dots) shows a spatio-temporal pattern where neighboring cells have a higher degree of correlation (red line, error bars are ±1SD). Blue line is a fitted exponential decay. (b) The distribution of STTC scores for each cell in greyscale. Spatio-temporal patterning is not present during stochastic activity. (c) The correlation of each cell to all other cells (black dots) shows no spatio-temporal pattern (red line, error bars are ±1 SD) and a lower degree of correlation than in embryos displaying synchronous events. Blue line is a fitted exponential decay. (d) The distribution of STTC scores for each cell displayed in greyscale.


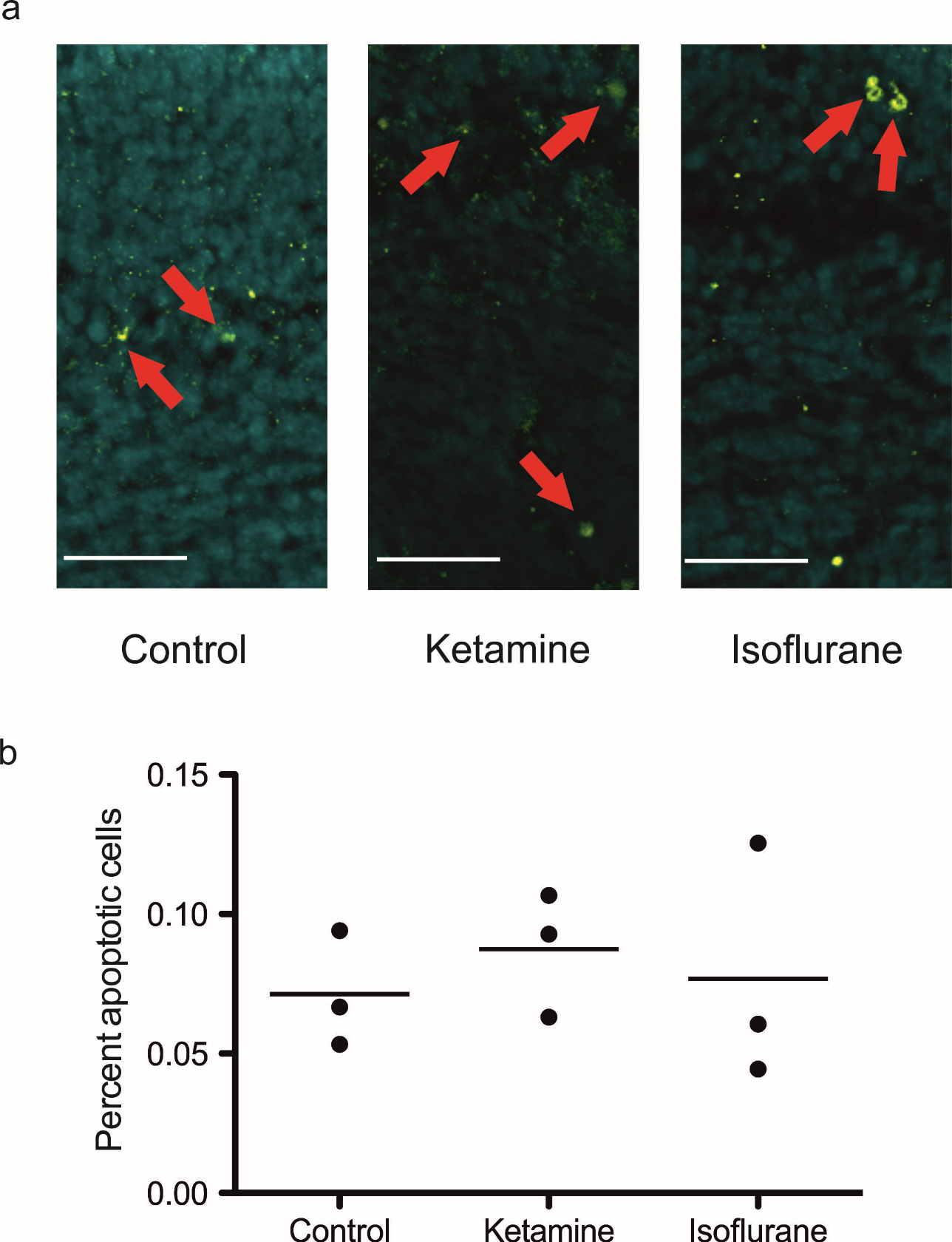


**Supplementary Figure 2. Effects of ketamine and isoflurane anaesthesia on cell death** (a) Cortical cryosections (20 µm) were stained with antibodies to apoptotic marker cleaved caspase-3 (Alexa 568, shown in green) from harvested E15 embryos after 3 hours of maternal anaesthesia at E14. Nuclei were stained with Hoechst 33342 (shown in cyan). Red arrows indicate apoptotic cells. Scale bars correspond to 20µm. (b) Quantification of apoptotic cells, percentage is normalized to density of cortical cells (n=3 embryos). Data are shown as mean.

**Supplementary videos**

**Supplementary video 1.** Spontaneous calcium activity in mouse embryonic cortex under isoflurane anaesthesia at E14. Field of view is 314×314 µm, playback rate is 50 fps.

**Supplementary video 2.** Baseline of calcium activity before the ketamine injection in mouse embryonic cortex under isoflurane anaesthesia at E15. Field of view is 528×536 µm, playback rate is 50 fps.

**Supplementary video 3.** Shut-down of calcium activity after the ketamine injection (1 µl, conc.50mg/ml) in mouse embryonic cortex under isoflurane anaesthesia at E15. Field of view is 512×512 µm, playback rate is 50 fps.

**Supplementary video 4.** Calcium activity after the vehicle injection (1 µl, 0.9% saline solution) in mouse embryonic cortex under isoflurane anaesthesia at E15. Field of view is 512×512 µm, playback rate is 50 fps.

**Supplementary video 5.** Cell motility before the ketamine injection in mouse embryonic cortex under isoflurane anaesthesia at E14. Field of view is 522×522 µm, playback rate is 96 fps.

**Supplementary video 6.** Ceasing of cell motility after the ketamine injection (1 µl, conc.50mg/ml) in mouse embryonic cortex under isoflurane anaesthesia at E14. Field of view is 512×512 µm, playback rate is 96 fps.

**Supplementary video 7.** Highly motile cell protrusions in mouse embryonic cortex under isoflurane anaesthesia at E14. Field of view is 126×126 µm, playback rate is 50 fps.

**Supplementary video 8.** Blocking of cell protrusions motility after the ketamine injection (1 µl, conc.50mg/ml) in mouse embryonic cortex under isoflurane anaesthesia at E14. Field of view is 126×126 µm, playback rate is 50 fps.
